# Demographic history shapes genomic ancestry in hybrid zones

**DOI:** 10.1002/ece3.7833

**Published:** 2021-07-05

**Authors:** Megan E. Frayer, Bret A. Payseur

**Affiliations:** ^1^ Laboratory of Genetics University of Wisconsin Madison Madison WI USA

**Keywords:** ancestry, genomics, hybrid zones, migration, speciation

## Abstract

Demographic factors such as migration rate and population size can impede or facilitate speciation. In hybrid zones, reproductive boundaries between species are tested and demography mediates the opportunity for admixture between lineages that are partially isolated. Genomic ancestry is a powerful tool for revealing the history of admixed populations, but models and methods based on local ancestry are rarely applied to structured hybrid zones. To understand the effects of demography on ancestry in hybrids zones, we performed individual‐based simulations under a stepping‐stone model, treating migration rate, deme size, and hybrid zone age as parameters. We find that the number of ancestry junctions (the transition points between genomic regions with different ancestries) and heterogenicity (the genomic proportion heterozygous for ancestry) are often closely connected to demographic history. Reducing deme size reduces junction number and heterogenicity. Elevating migration rate increases heterogenicity, but migration affects junction number in more complex ways. We highlight the junction frequency spectrum as a novel and informative summary of ancestry that responds to demographic history. A substantial proportion of junctions are expected to fix when migration is limited or deme size is small, changing the shape of the spectrum. Our findings suggest that genomic patterns of ancestry could be used to infer demographic history in hybrid zones.

## Graphical abstract

Ancestry changes along admixed chromosomes have been used by numerous models and methods to reveal the history of admixed populations, yet these approaches are rarely applied to structured hybrid zones. In this study, we perform simulations under a stepping‐stone model, focusing on migration rate, deme size, and hybrid zone age as key parameters. We find that ancestry patterns including our novel summary—the junction frequency spectrum—are sensitive indicators of demographic history.

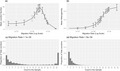

## 1 INTRODUCTION

During speciation, lineages independently accumulate genetic variation. When these lineages come back into contact and mate, certain combinations of mutations may reduce the fitness of hybrids and confer barriers to reproduction between the lineages. If a balance is reached between migration and selection against hybrids in the region of contact, a stable population structure can form, with demes containing individuals of mixed ancestry bridging the gap between the original lineages.

Migration across this metapopulation, known as a hybrid zone, shapes its dynamics by controlling the flow of alleles. Migration tends to homogenize allele frequencies between demes (Wright, 1931), slowing genomic divergence. Restricting migration in a subdivided metapopulation can facilitate local adaptation, whereas demes begin to behave as a single panmictic population when migration is high (Barton & Whitlock, 1997; Maruyama & Kimura, 1980; Whitlock & Barton, 1997). Hybrid zones may additionally feature migration from source populations, which replenishes chromosomes and combinations of alleles found outside of the region of contact (Barton, 1979a; Feldman & Christiansen, 1974; Harrison, 1990). Stable hybrid zones are often described using a tension zone model, under which the balance between migration across the zone and selection against hybrids maintains sigmoidal clines in allele frequencies (Barton, 1979a; Barton & Hewitt, 1985). In this scenario, migration works in direct opposition to selection to establish a hybrid zone structure.

The dynamics of a hybrid zone are also governed by genetic drift. Drift increases the variance in allele frequency among demes, leading to steeper clines, even at neutral loci (Polechová & Barton, 2011). In addition, drift reduces the efficacy of selection in small populations (Kimura et al., 1963). Drift may be a strong force in hybrid zones because there are reasons to suspect that hybrid populations will often be small. If selection against hybrids is strong, low hybrid fitness could limit population growth. Hybrid zones generally occur at the edges of species ranges, which can have low population densities (Bridle & Vines, 2007). Furthermore, range edges can be highly fragmented (Bridle & Vines, 2007), which may reduce migration and strengthen drift. Finally, drift is a potential explanation for instances of variable hybridization outcomes across unique meetings of the same species (Mandeville et al., 2017).

The genomic pattern underlying the observed genetic variation in a hybrid zone is ancestry. Over time, the configuration of ancestry along chromosomes changes as meiotic recombination breaks down segments inherited from each lineage. This expectation has spurred the creation of methods that use inferred ancestry to estimate admixture time (Corbett‐Detig & Nielsen, 2017; Liang & Nielsen, 2014; Medina et al., 2018; Moorjani et al., 2011; Pool & Nielsen, 2009). Migration generally acts on ancestry in opposition to recombination and time, by replacing some of the chromosomes that were previously shuffled by recombination. Drift affects the rate at which ancestry patterns are fixed or lost from a hybrid population. Incorporating drift into analytical models better captures the behavior of ancestry in admixed populations (Gravel, 2012).

Patterns of local ancestry in individuals can be described by inferring the genomic locations of junctions. Junctions are transition points between tracts of alternative ancestries along a chromosome, first used by Fisher (1949, 1954) to model the effects of inbreeding. Junctions are formed by recombination events between chromosomes with different ancestries and are inherited like point mutations (Fisher, 1954). Junctions can be counted, and the distances between junctions (“tract lengths”) can be measured. The distribution of tract lengths is the inverse of the distribution of junction density.

Junction density and tract length respond to demographic history and selection (Gravel, 2012; Hvala et al., 2018; Janzen et al., 2018; Pool & Nielsen, 2009). Junction density increases as tracts shorten over time (Liang & Nielsen, 2014; Pool & Nielsen, 2009). In an isolated population, junction density approaches an equilibrium value, and the population converges on a single ancestry pattern (Chapman & Thompson, 2002). Analytical models show that migration leads to longer tract lengths (Gravel, 2012; Pool & Nielsen, 2009). In a hybrid swarm model, increasing population size raises the rate of junction formation and the time required to reach the maximum number of junctions (Janzen et al., 2018).

Although ancestry‐based approaches are increasingly used to reconstruct demography in humans (Bryc et al., 2010; Bycroft et al., 2019; Hellenthal et al., 2014) and other species (Lavretsky et al., 2019; Leitwein et al., 2018), their application to hybrid zones between divergent lineages remains limited compared with analyses of allele frequency clines (Gompert et al., 2017; Payseur & Rieseberg, 2016). Junction‐based methods have been applied to understand hybrid speciation (Buerkle & Rieseberg, 2008; Ungerer et al., 1998), but not in structured hybrid zones. Part of the explanation for this deficit is a lack of specific theoretical predictions for ancestry in hybrid zones with realistic population structure. Existing analytical models of ancestry necessarily make important simplifying assumptions. Chapman and Thompson (2002) assumed a single pulse of admixture, whereas Pool and Nielsen (2009) considered an island model of migration. In a spatially explicit model of admixture, Sedghifar et al. (2015) derived useful expressions for tract length as a function of individual dispersal distance and time since initial contact, but ignored genetic drift. Hvala et al. (2018) simulated ancestry in a stepping‐stone framework that considered drift, but explored a limited part of the demographic parameter space.

Given the growing success of ancestry‐based frameworks for interpreting genomic data and the insights that hybrid zones provide about speciation, we examined how demographic history affects ancestry in a structured hybrid zone. We performed neutral simulations under a stepping‐stone model, emphasizing the effects of time, gene flow and drift on the dynamic behavior of ancestry junctions, and heterogenicity (the heterozygosity of ancestry). Our results highlight the sensitivity of ancestry to demography and motivate the application of ancestry‐based methods to infer history in real hybrid zones.

## 2 MATERIALS AND METHODS

Individual‐based, forward‐in‐time simulations were run using *forqs* (Kessner & Novembre, 2014). This program records recombination events and tracks the founding haplotypes of a population through time. By starting with two haplotypes that represent two source populations, the resulting haplotype blocks can be used to follow ancestry and to determine which recombination events are also ancestry junctions. We analyzed these haplotypes directly, bypassing the generation of nucleotide sequences and assuming perfect knowledge of ancestry.

**FIGURE 1:**
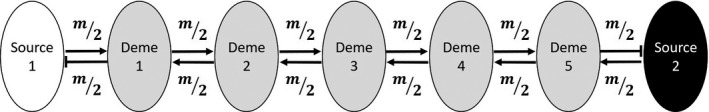
Stepping‐stone model assumed in individual‐based simulations. Five hybrid populations (demes) exchange migrants at the same rate. Source populations contribute to the hybrid demes but do not receive migrants

We simulated a hybrid zone based on the stepping‐stone model (Feldman & Christiansen, 1974; Kimura & Weiss, 1964). The stepping‐stone model is often used to describe hybrid zones (Barton, 1979b; De La Torre et al., 2015; Dudek et al., 2019; Gavrilets, 1997), and it better captures spatial dynamics than the Wright–Fisher model of admixture that has been employed to examine ancestry in admixed populations (Gravel, 2012; Liang & Nielsen, 2014). We used the “LinearSteppingStone” configuration in *forqs*, which generates a string of subpopulations (“demes”) connected by migration. We modeled five hybrid demes connecting two source populations that remained unadmixed (Figure 1). At generation 0, the two source populations were established. Then, the hybrid zone was sequentially filled by individuals from the source populations over the next few generations, and the central hybrid population was formed in generation 4 as a 50/50 mix. Consequently, the first phase of each simulation consisted of initial admixture and eventual loss of parental individuals from the hybrid demes.

At the establishment of each deme, the number of individuals was fixed at a given deme size. Because we used an individual‐based simulator, this deme size was the actual number of individuals generated in that deme. All individuals were equally likely to contribute to the next generation.

Individuals reproduced as diploid hermaphrodites. We followed the fate of a single pair of 1 Megabase (Mb) chromosomes. In each nonoverlapping generation, chromosomes completed meiosis. A Poisson‐distributed number of crossovers was generated, and these crossovers were placed along the chromosomes at random positions drawn from a uniform distribution. Generations were treated as the unit of time, and our results could be applied to organisms with any generation time. For simplicity, we assumed a recombination rate of 0.51 cM/Mb, equal to the genomic average for house mice (Cox et al., 2009), a classic genetic and genomic model for studying reproductive isolation in hybrid zones (Boursot et al., 1993; Sage et al., 1993; Teeter et al., 2010; Tucker et al., 1992; Turner et al., 2014). Because we focused on the effects of demography, natural selection was absent from all simulations. Migration occurred with the same rate across demes, with only neighboring demes exchanging migrants (Figure 1). We assumed no migration back into the source populations; half of the migrants chosen from the outermost hybrid demes were discarded, rather than being transferred to the source populations.

From each simulation, 18 individuals were sampled from the central population. We chose this number as a practical sample size for studies of real hybrid zones, where determination of fine‐scale ancestry will typically require whole genome sequencing. One hundred replicates were run for each combination of parameters.

For comparison, simulations were also run under a different model with only one hybrid population receiving migrants from two source populations, hereafter referred to as the “hybrid swarm” model. These simulations were run using the same framework in *forqs*, but with only one hybrid “deme” filled in the first generation of the simulation.

### 2.1 Demography

The stepping‐stone model features several parameters that could affect the dynamics of hybridization. We focused on deme size, migration rate, and time since hybrid zone formation. We conducted simulations for several values of each parameter (Table 1) to determine how these parameters shape genomic ancestry patterns. We chose an initial set of values to cover a broad parameter space and subsequently added values to further clarify observed patterns. Ranges of parameter values were chosen with actual hybrid zones in mind.

**TABLE 1:** Tested values of demographic parameters

Parameters	Values tested
Deme size	100; 500; 1,000; 3,000; 5,000
Generations of admixture	100; 500; 1,000; 2,000; 4,000; 6,000; 8,000; 10,000; 12,000; 14,000; 16,000; 18,000; 20,000; 22,000; 24,000; 26,000; 28,000; 32,000; 36,000 40,000; 44,000; 48,000; 52,000; 56,000; 60,000
Migration rate	0; 1e−8; 1e−7; 1e−6; 1e−5; 2e−5; 4e−5; 6e−5; 8e−5; 1e−4; 2e−4; 4e−4; 6e−4; 8e−4; 1e−3; 1e−2
Recombination rate (cM/Mb)	0.51
Deme number	1; 5

For a subset of parameter combinations, we explored a case without migration. These simulations were run in the stepping‐stone framework described above, but once all demes were established, migration between them was eliminated. This approach enabled direct comparison to simulations of the stepping‐stone model. These simulations were used in conjunction with simulations of the hybrid swarm model to better understand behavior under the stepping‐stone model, as well as to make direct comparison to existing analytical predictions.

### 2.2 Summary statistics

The output of simulations contained ancestry information about each of the two chromosomes in an individual. Nevertheless, recognizing the many challenges associated with reconstructing haplotype phase in hybrid zones, we focused on summary statistics that could be obtained from unphased data.

**FIGURE 2:**
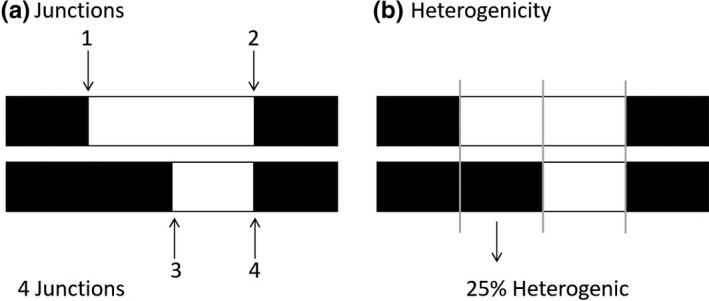
Descriptors of ancestry. (a) Heterogenicity is the proportion of the genome that is heterozygous for ancestry. (b) Junctions are transition points between ancestries along chromosomes

Summary statistics were chosen to reflect basic patterns of ancestry. First, junction number was counted in an individual as the number of switch points between ancestries (Figure 2a) (Fisher, 1954). Second, heterogenicity was computed as the proportion of an individual genome harboring ancestry from both source populations (i.e., different ancestries on the two chromosomes; Figure 2b) (Fisher, 1954). Third, we tabulated the frequency of each junction across the sample, thereby generating a “junction frequency spectrum” analogous to the site frequency spectrum commonly used to describe single nucleotide polymorphisms (Braverman et al., 1995; Gutenkunst et al., 2009; Tajima, 1989). Because junctions only form when hybridization has occurred, each junction can be considered derived relative to the ancestral state of having no junction. Thus, we treated the junction frequency spectrum as unfolded. Because junctions can be inferred even when they are invariant in the sample (and the population), we included fixed junctions in the frequency spectrum.

For junction number and heterogenicity, we computed the mean, median, variance, and skew across the 18 sampled individuals. For junction number, we also computed the total count in the sample. We examined the junction frequency spectrum graphically, representing each frequency category as the proportion of junctions found a given number of times in the sample. We further characterized the junction frequency spectrum by computing the number of singleton junctions (those occurring on only one chromosome in the sample), the proportion of singleton junctions, the number of unique junctions (the number of independently occurring junctions, regardless of their frequency), and the skew of the spectrum.

All simulation scripts, input files, and results from this study have been deposited on Dryad (https://doi.org/10.5061/dryad.3tx95x6gk).

## 3 RESULTS

### 3.1 Summary statistics

Results for all summary statistics of ancestry we computed are available on Dryad (https://doi.org/10.5061/dryad.3tx95x6gk). Here, we focus on results for mean junction number, mean heterogenicity, and the junction frequency spectrum. These summary statistics were the most sensitive to demography across the parameter space we surveyed.

### 3.2 Ancestry over time

**FIGURE 3:**
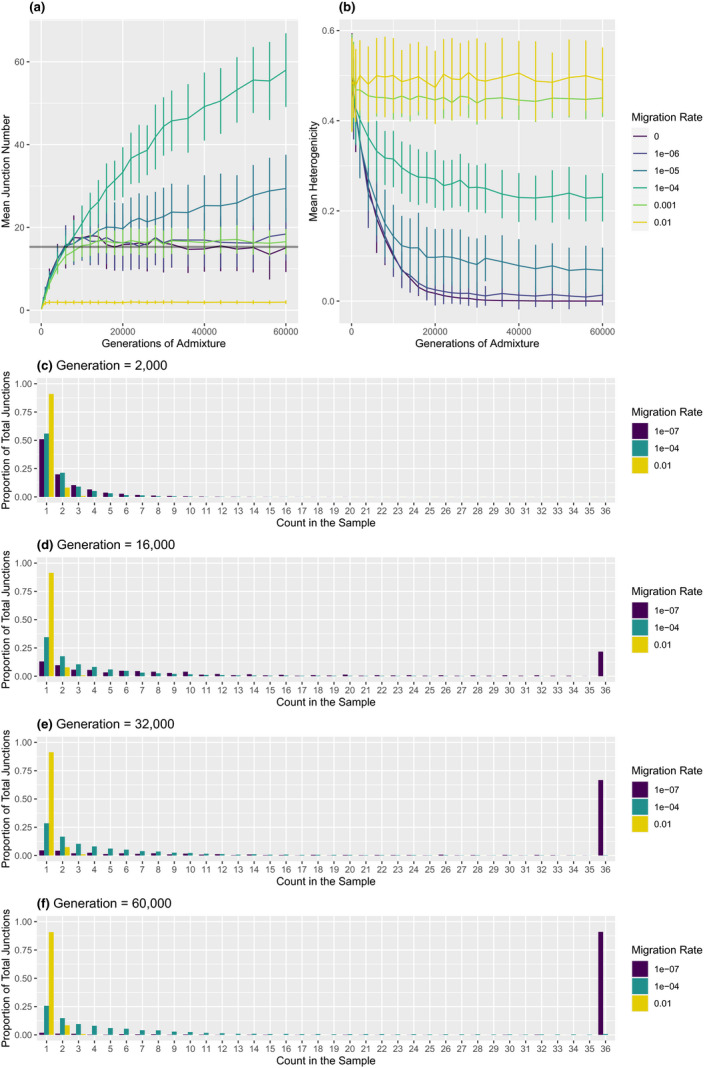
Ancestry over time. In simulations with a deme size of 3,000, mean junction number in the sample (a) and mean heterogenicity in the sample (b) are shown as the mean of 100 replicates. Bars represent one standard deviation above or below the mean. Average junction frequency spectra are shown for three migration rates at generation 2,000 (c), 16,000 (d), 30,000 (e), and 60,000 (f). In panel A, the gray line represents the expected number of junctions for the no‐migration case based on Janzen et al. (2018)

The first demographic parameter we examined was time since hybrid zone formation, measured in generations. Because our simulations began with two separate populations, the hybrid zone was established in the initial phase of the simulations. For most parameter combinations (all but the highest migration rate and the smallest deme size), the central population samples are nearly all hybrids by the 500th generation, so we focus on timepoints after 500 generations. Junctions are expected to accumulate over time, approaching an equilibrium that reflects a balance between migration, recombination, and drift (Chapman & Thompson, 2002; Hvala et al., 2018), and our findings match that expectation (Figure 3a). For simulations without migration, we found that the mean junction number at equilibrium matches predictions from analytical theory (Janzen et al., 2018).

Heterogenicity decreases over time, also approaching an equilibrium value (Figure 3b). In most cases, it appears that heterogenicity reaches equilibrium before junction number. For example, when the migration rate is 0.001 and the deme size is 3,000, junction number settles between 12,000 and 14,000 generations, whereas heterogenicity barely changes between 6,000 and 8,000 generations.

The shape of the junction frequency spectrum (hereafter denoted as JFS) changes over time. The proportion of singletons decreases and the tail of the distribution lengthens, indicating that junctions are rising to higher frequencies (Figure 3c‐f). The JFS approaches equilibrium near the same time as junction number.

### 3.3 Effects of migration on ancestry

The second demographic parameter we considered was migration rate. The migration rate modifies both the time to equilibrium and the equilibrium values for junction number and heterogenicity (Figure 3a,b).

**FIGURE 4:**
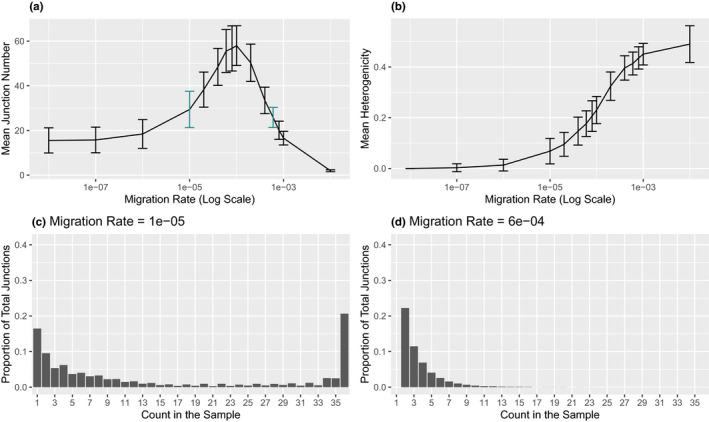
Impact of migration on ancestry. In simulations with a deme size of 3,000 at generation 60,000, mean junction number in the sample (a) and mean heterogenicity in the sample (b) are shown as the mean of 100 replicates. Bars represent one standard deviation above or below the mean. Average junction frequency spectra are shown for the colored points: a migration rate of 0.00001 (c) and 0.0006 (d), which have similar mean junction numbers (29.4 and 25.8, respectively)

While we expected more migration to decrease equilibrium junction number due to the addition of unadmixed chromosomes (from source populations), we find that the relationship is more complicated (Figure 4a). Changes in the level of migration can increase or decrease the number of junctions. Very high migration reduces junction numbers compared with the case of no migration, whereas very low migration and no migration result in similar junction numbers. However, in between these extremes, there is a zone where increasing migration raises the number of junctions (relative to no migration). Small changes in migration rate can generate substantial effects. For example, samples simulated with an intermediate migration rate of 0.0001 (and a deme size of 3,000) have an average of 57.9 junctions at generation 60,000 and are still adding junctions, whereas samples simulated with the similar migration rate of 0.0004 stop accumulating junctions at an average of 33.5 near generation 34,000.

The effect of migration rate on heterogenicity is more straightforward than the effect on junction number (Figure 4b). Without migration, heterogenicity goes to zero over time as observed for an admixed Wright–Fisher model (Chapman & Thompson, 2002), but with migration heterogenicity decreases to a nonzero equilibrium value. With migration, heterogenicity is lost more slowly than the exponential decay predicted for a population with no migration (Chapman & Thompson, 2002).

Decreasing migration increases junction frequencies, lengthening the tail of the JFS (Figure 4c,d). No migration or very low migration leads to a high proportion of fixed junctions, producing a spectrum that is u‐shaped or even right‐skewed. The occurrence of fixed junctions is a strong indicator of limited migration. For a deme size of 3,000, no fixed junctions are found in any simulation with a migration rate above 0.0004, and fixed junctions are rare at migration rates between 0.0001 and 0.0004.

When migration rates are very low, all junctions may eventually fix (as in the case of no migration) or an equilibrium featuring a mixture of polymorphic and fixed junctions might be reached. Populations simulated with a migration rate of 0.000 001 (and a deme size of 3,000) appear to achieve an equilibrium in which 68% of junctions are fixed and populations simulated with a migration rate of 0.000 01 (and a deme size of 3,000) appear to achieve an equilibrium in which 19% of junctions are fixed.

### 3.4 Effects of deme size on ancestry

**FIGURE 5:**
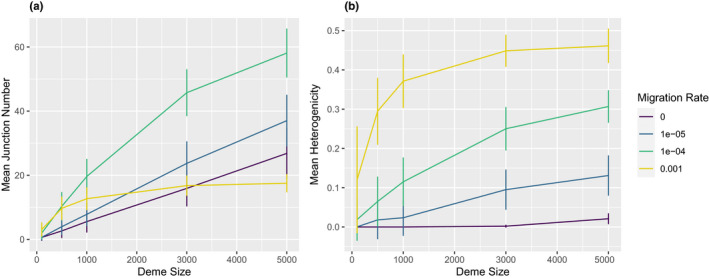
Impact of deme size on junction number and heterogenicity. In simulations at generation 30,000, mean junction number in the sample (a) and mean heterogenicity in the sample (b) are shown as the mean of 100 replicates. Bars represent one standard deviation above or below the mean

**FIGURE 6:**
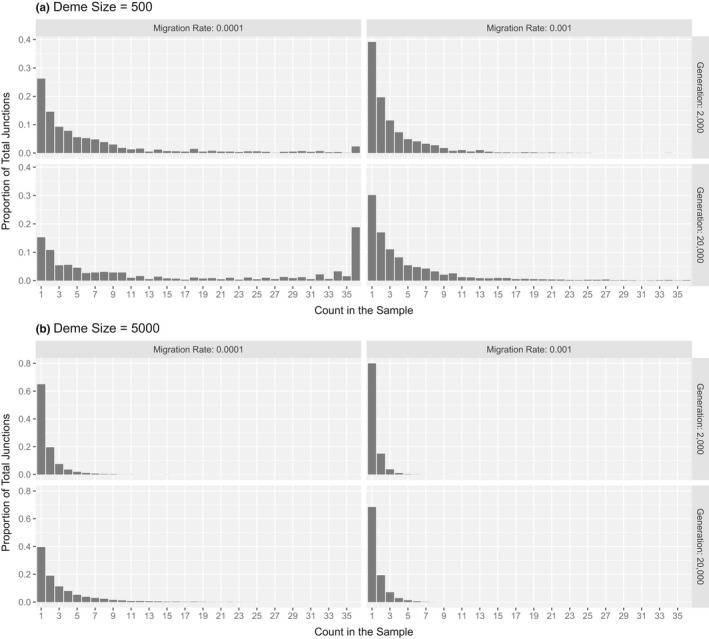
Impact of deme size on the junction frequency spectrum. Average junction frequency spectra are shown for a deme size of 500 (a) and 5,000 (b) for two migration rates and two timepoints

The last demographic parameter we explored was deme size. Increasing deme size generally increases the number of junctions (Figure 5a) as well as the heterogenicity (Figure 5b). Deme size strongly affects the shape of the JFS (Figure 6). Small demes harbor relatively more junctions at higher frequencies (a longer tail) and, in some cases, a high proportion of fixed junctions (a u‐shaped spectrum).

**FIGURE 7:**
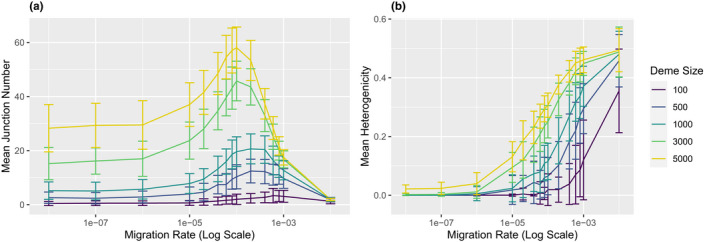
Interactions between migration rate and deme size impact ancestry patterns. In simulations at generation 30,000, mean junction number in the sample (a) and mean heterogenicity in the sample (b) are shown as the mean of 100 replicates. Bars represent one standard deviation above or below the mean

The effects of deme size depend on migration rate (Figures 5a,b and 7). Increasing deme size enhances the potential for junction accumulation at intermediate and low migration rates (Figure 7a). In other words, junction numbers are less affected by changes in deme size when migration is common. This pattern may be driven by higher heterogenicity in larger demes (Figure 7b).

### 3.5 The effect of population structure

**FIGURE 8:**
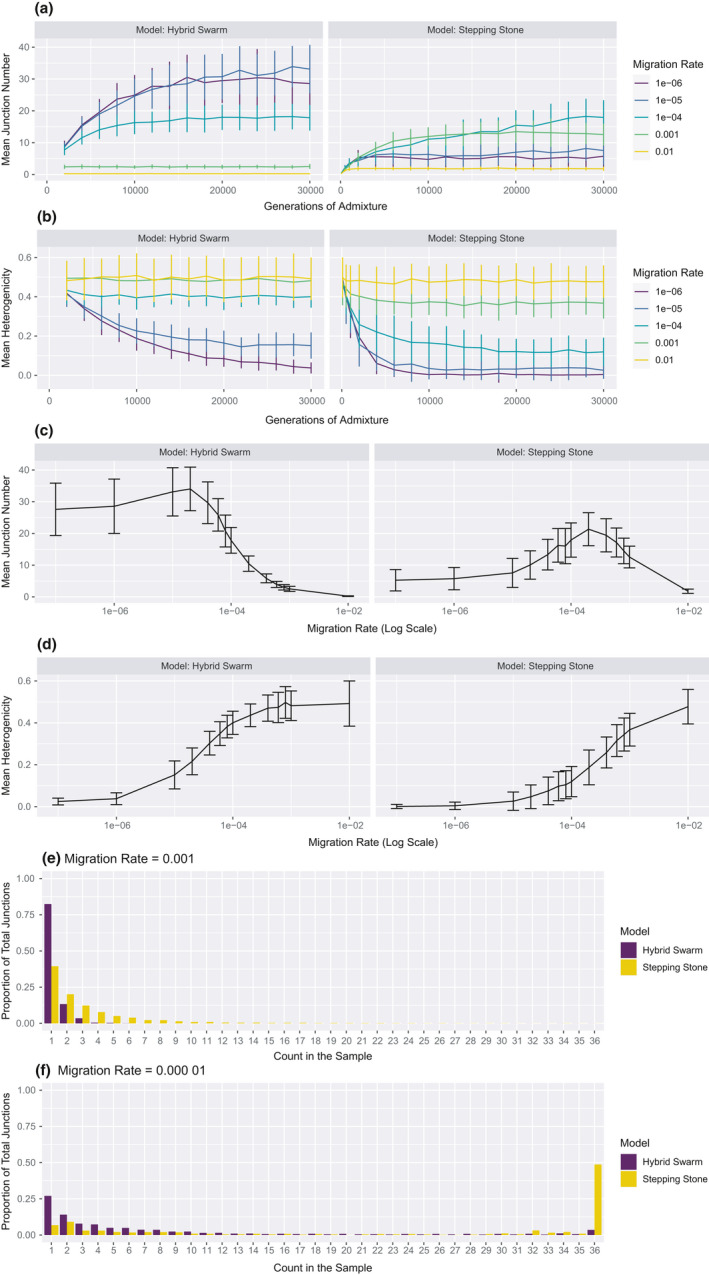
Comparison of ancestry patterns from hybrid swarm and stepping‐stone models. For simulations at several migration rates with a total hybrid population of 5,000 individuals, mean junction number in the sample (a) and mean heterogenicity in the sample (b) are shown as the mean of 100 replicates. In simulations at generation 30,000 and a total hybrid population of 5,000 individuals, mean junction number in the sample (c) and mean heterogenicity in the sample (d) are shown as the mean of 100 replicates. Bars represent one standard deviation above or below the mean. Average junction frequency spectra are shown for a high migration rate (e) and a low migration rate (f) for both models at generation 30,000 with a total hybrid population of 5,000 individuals

While deme size is a convenient proxy for effective population size, deme number and migration rate also influence effective population size in a stepping‐stone model (Barton & Whitlock, 1997; Whitlock & Barton, 1997). To better understand the impact of population structure on ancestry patterns, we conducted simulations under a “hybrid swarm” model, with only one hybrid deme receiving migrants from the source populations. We directly compared simulations with the same total hybrid population size: Hybrid swarm simulations had a single deme of 5,000 individuals and stepping‐stone simulations had five demes with 1,000 individuals in each deme (Figure 8). While the results follow many of the same trends, it is clear that population structure affects ancestry patterns. For example, increasing migration in a stepping‐stone model can raise the junction number more than in the hybrid swarm model (Figure 8c). Furthermore, the stepping‐stone model allows junctions to increase in frequency more than the hybrid swarm model, producing a relatively right‐skewed junction frequency spectrum (Figure 8e,f). These patterns suggest that results from a hybrid swarm model will be difficult to generalize to populations with the type of structure that characterizes natural hybrid zones.

## 4 DISCUSSION

Hybrid zones test the progression of speciation between diverging lineages, potentially leading to reinforcement of reproductive barriers (completion of speciation) or to fusion (reversal of speciation) (Abbott et al., 2013; Coyne & Orr, 2004). The study of hybrid zones provides insights into these alternative outcomes as well as the genetic and evolutionary processes that drive speciation in general (Barton & Hewitt, 1985; Harrison, 1990). Demographic factors are key contributors to both the speciation process and the dynamics of hybrid populations. The intensity and spatial pattern of gene flow between populations depend on migration. The ability of hybrid populations to persist and the effectiveness of selection within them are determined by deme size. As the approach of using genomic patterns observed in natural hybrids to identify the incompatible mutations that isolate nascent species continues to grow in popularity (Gompert et al., 2017; Payseur & Rieseberg, 2016), it is important to consider how demography alters hybrid genomes.

Our simulations reveal that migration and deme size combine to leave detectable footprints in patterns of ancestry in hybrid zones. A novel metric, the junction frequency spectrum (JFS), illustrates how competition between gene flow and drift (controlled by the interactions between migration and deme size) dictates the dynamics of ancestry in the genome. Increasing migration reduces junction accumulation, shortening the tail of the junction frequency spectrum. With little migration, drift within demes fixes junctions. At intermediate migration rates and after enough time, junction frequencies arrive at equilibria reflecting a balance between gene flow and drift. Collectively, these observations indicate strong connections between migration, population size, and the shape of the junction frequency spectrum.

Our findings also demonstrate that the relationship between demographic parameters and ancestry can differ depending on parameter values. Although previous work showed that increasing migration reduces junction number (Hvala et al., 2018) (or equivalently, expands ancestry tract length; Gravel, 2012), we found that higher migration can lead to *more* junctions when migrants are too infrequent to prevent junction accumulation but still frequent enough to contribute to the genetic variation present in the deme. Migrants are likely to carry variants that have drifted to low frequencies in central demes, due to the independent effects of drift across hybrid demes (Barton & Whitlock, 1997). This diversity allows the central deme to maintain a higher level of heterogenicity than it would without migration. Heterogenicity is the substrate for junction formation. This effect is present but much weaker in the simulations under the hybrid swarm model, likely because all migrant chromosomes are unadmixed, narrowing the window between migration increasing heterogenicity and swamping out junctions that have been formed in the hybrids.

The role of demographic history in shaping ancestry patterns was examined in previous theoretical work. Analyzing an isolated hybrid population, Janzen et al. (2018) found that smaller population size, biased starting ratios of ancestries, and nonuniform recombination all slow the formation of junctions by reducing heterogenicity. Modeling an admixture zone over continuous space, Sedghifar et al. (2015) reported that including nearest‐neighbor migration impedes the decay of admixture linkage disequilibrium, likely due to the repeated introduction of unadmixed chromosomes from the periphery of the population. By jointly considering gene flow and drift in a stepping‐stone model, our study complements Janzen et al. (2018) (which ignored gene flow) and Sedghifar et al. (2015) (which ignored drift). In addition to observing separate effects of gene flow and drift that qualitatively match those in Janzen et al. (2018) and Sedghifar et al. (2015), we demonstrate that these two processes interact to shape ancestry.

Our conclusions are accompanied by caveats and opportunities for extension. First, we expect our assumption of neutrality to be violated in most hybrid zones between divergent lineages, at least for those parts of the genome responsible for reproductive isolation. Selection against hybrids distorts allele frequency clines (Barton, 1979a; Payseur, 2010) and maintains longer ancestry tracts with fewer junctions than expected under neutrality (Baird et al., 2003; Barton, 1983; Hvala et al., 2018; Sedghifar et al., 2016), suggesting that the effects of demography we documented should be examined in models with selection. The effect of selection is likely to vary across demographic histories. In several of the scenarios examined here, drift is strong and may readily overcome the effects of selection. Strong drift drives genetic patterns in some natural hybrid zones (e.g., McFarlane et al., 2021) and may be particularly relevant in zones where the hybrid populations are small or patchy.

Although the stepping‐stone model we studied captures important aspects of hybrid zone structures, actual hybrid zones can take a variety of forms. One example is a mosaic or patchy population structure (Harrison & Rand, 1989). Depending on the connections between mosaic hybrid populations and source populations, these types of hybrid zones could be even more strongly affected by drift, leading to a higher proportion of common junctions over time. There can also be variation in the relative rates of migration from each of the source populations (*e.g*., Field et al., 2011). In these situations, it is possible that other metrics, such as ancestry proportion, would be stronger indicators of the migration rate. We might expect to see heterogenicity deflated due to a bias toward one parental type, leading to a decrease in junction formation, as seen in an isolated hybrid population (Janzen et al., 2018). Based on our results, considering the population structure of a given hybrid zone will be critical to interpreting its ancestry patterns.

Hybrid zones are dynamic. Our simulations assumed that deme sizes and migration rates are constant over long periods of time, an assumption that is likely to be violated in natural hybrid zones (Barton, 1979b; Buggs, 2007; Wielstra, 2019). The possibility that demographic parameters vary over time should be considered when interpreting ancestry patterns from hybrid zones.

Recombination produces junctions, suggesting that recombination rate shapes the ancestry signatures that demography leaves along chromosomes. We assumed that crossovers appear at a single rate, independently of one another. Variation in recombination rate along a chromosome (Haenel et al., 2018; Nachman, 2002; Yu et al., 2001) as well as crossover interference (Berchowitz & Copenhaver, 2010)—both widespread phenomena—should further increase heterogeneity in junction patterns conferred by demography.

Despite these caveats, our findings emphasize the potential for using ancestry patterns to reconstruct demographic history in hybrid zones. Existing statistical methods enable the probabilistic inference of fine‐scale ancestry switching along chromosomes from genomic data (Baran et al., 2012; Browning & Browning, 2011; Corbett‐Detig & Nielsen, 2017; Guan, 2014; Price et al., 2009; Wegmann et al., 2011). Ancestry patterns in admixed populations have often been used to pinpoint the timing of initial gene flow, especially in humans (Corbett‐Detig & Nielsen, 2017; Hellenthal et al., 2014; Henn et al., 2012; Liang & Nielsen, 2014; Moorjani et al., 2011; Patterson et al., 2012). In contrast, few analytical frameworks have been developed to characterize demographic history in populations with structures typical of hybrid zones. This gap is surprising, given that gene flow is usually the primary subject of interest when students of speciation examine hybrid zones. In addition, the effective population size of a metapopulation is shaped by both deme size and migration (Maruyama & Kimura, 1980; Whitlock & Barton, 1997). Hybrid zones with smaller demes, less migration, or both are expected to experience more drift. These ideas suggest that the common practice of ignoring population size when drawing evolutionary inferences from hybrid zones in the context of speciation could be misleading.

By identifying summary statistics that are sensitive to migration rate and population size, we have taken a first step toward developing an analytical framework for the reconstruction of demographic history from genomic data in hybrid zones. We view the junction frequency spectrum as an especially informative summary of ancestry. Inference of demographic history could follow two paths. First, simulation results such as ours could be used to guide mathematical theory that connects junction patterns to demographic parameters, leading to formulae that could be used for parameter estimation. For example, the junction frequency spectrum appears to follow an exponential distribution under a range of conditions. Second, inference could proceed by searching by simulation for parameter combinations that produce similar junction patterns to those observed in hybrid zone data, through Approximate Bayesian Computation or related approaches. To mitigate effects of linked selection on inference, genomic regions with few genes and high recombination rates could be chosen. The reconstruction of demographic history could provide a baseline for detecting selection by scanning genomes from hybrid zones. As genomic datasets from hybrid zones become more readily available, the inference of demographic history will be an important step toward understanding the dynamics of hybrid zones and the process of speciation.

## CONFLICT OF INTEREST

The authors declare no conflict of interest.

## AUTHOR CONTRIBUTIONS

**Megan E. Frayer:** Conceptualization (equal); Data curation (lead); Formal analysis (lead); Investigation (lead); Methodology (lead); Project administration (equal); Resources (equal); Software (lead); Validation (lead); Visualization (lead); Writing‐original draft (lead); Writing‐review & editing (lead). **Bret A. Payseur:** Conceptualization (equal); Funding acquisition (lead); Methodology (supporting); Project administration (equal); Resources (equal); Supervision (lead); Writing‐review & editing (supporting).

## Data Availability

The scripts and data from this study have been deposited on Dryad (https://doi.org/10.5061/dryad.3tx95x6gk).
